# What exactly does the *Pf*K13 C580Y mutation in *Plasmodium falciparum* influence?

**DOI:** 10.1186/s13071-023-06024-4

**Published:** 2023-11-16

**Authors:** Wenwen Si, Yuemeng Zhao, Xixi Qin, Yixuan Huang, Jing Yu, Xiao Liu, Yanna Li, Xiaoli Yan, Qingfeng Zhang, Jun Sun

**Affiliations:** School of Medicine, Tongji University, Shanghai, People’s Republic of China

**Keywords:** Artemisinin resistance, *Pf*K13 mutation, *P. falciparum* 3D7^C580Y^, Transcriptome, Iron supply, Merozoite number, Hemozoin, Haem

## Abstract

**Background:**

The emergence and spread of artemisinin resistance threaten global malaria control and elimination goals, and encourage research on the mechanisms of drug resistance in malaria parasites. Mutations in *Plasmodium falciparum* Kelch 13 (*Pf*K13) protein are associated with artemisinin resistance, but the unique or common mechanism which results in this resistance is unclear.

**Methods:**

We analyzed the effects of the *Pf*K13 mutation on the transcriptome and proteome of *P. falciparum* at different developmental stages. Additionally, the number of merozoites, hemozoin amount, and growth of *P. falciparum* 3D7^C580Y^ and *P. falciparum* 3D7^WT^ were compared. The impact of iron supplementation on the number of merozoites of *P. falciparum* 3D7^C580Y^ was also examined.

**Results:**

We found that the *Pf*K13 mutation did not significantly change glycolysis, TCA, pentose phosphate pathway, or oxidative phosphorylation, but did reduce the expression of reproduction- and DNA synthesis-related genes. The reduced number of merozoites, decreased level of hemozoin, and slowed growth of *P. falciparum* 3D7^C580Y^ were consistent with these changes. Furthermore, adding iron supply could increase the number of the merozoites of *P. falciparum* 3D7^C580Y^.

**Conclusions:**

These results revealed that the *Pf*K13 mutation reduced hemoglobin ingestion, leading to artemisinin resistance, likely by decreasing the parasites' requirement for haem and iron. This study helps elucidate the mechanism of artemisinin resistance due to *Pf*K13 mutations.

**Graphical Abstract:**

**Figure:**
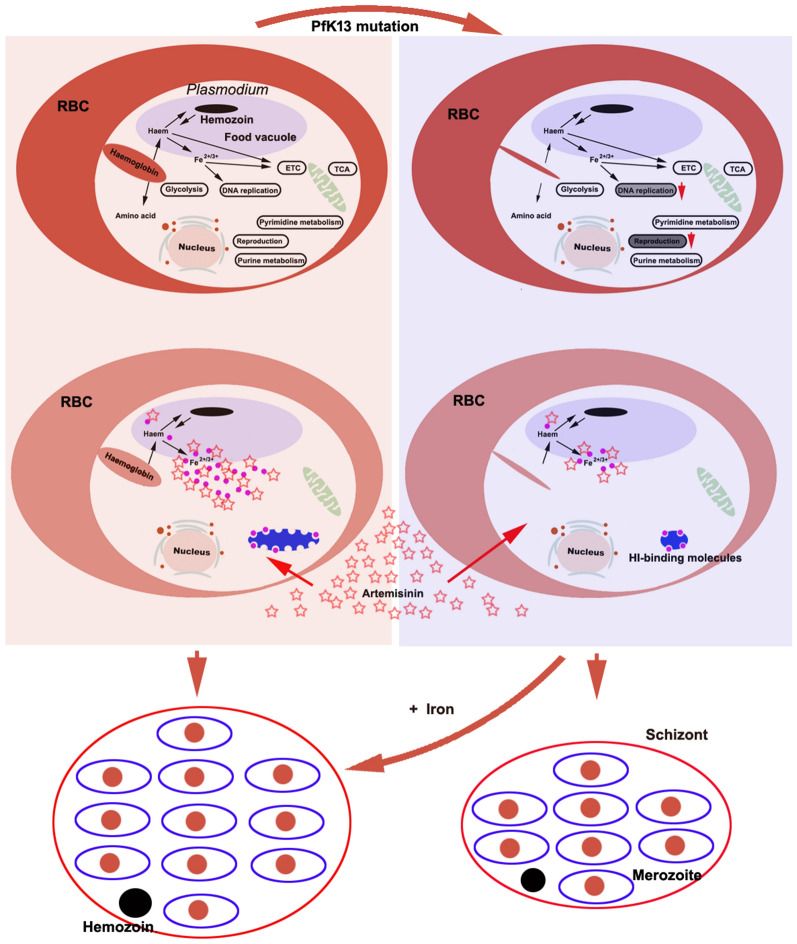

**Supplementary Information:**

The online version contains supplementary material available at 10.1186/s13071-023-06024-4.

## Background

The emergence and spread of artemisinin resistance endanger worldwide malaria control and elimination goals [1–4]. Although the mechanism of artemisinin resistance remains a subject of debate, numerous mutations in the *Plasmodium falciparum* Kelch 13 (*Pf*K13) protein have been associated with artemisinin resistance [5–8]. Frequently occurring mutations of *Pf*K13 include C580Y, R539T, I543, and Y493Y, with the C580Y mutation becoming increasingly dominant in the Greater Mekong subregion [9]. *Pf*K13 is located in the cytostome (cell mouth), through which the parasite takes up hemoglobin [10], the uptake and digestion of which likely determine artemisinin activity [11]. *Pf*K13 mutation likely confers artemisinin resistance by dampening hemoglobin endocytosis and artemisinin activation [8, 10]. Although malaria artemisinin resistance is thought to be associated with different *Pf*K13 mutations and relevant genetic and physiological backgrounds [12, 13], not all artemisinin resistance is due to the *Pf*K13 mutation [13–18]. Some parasites lacking *Pf*K13 mutations were still parasitaemic on day 3 after treatment with artemisinin derivatives [15], thus, the *Pf*K13 mutations that reduced hemoglobin endocytosis and led to decreased artemisinin activation were not the sole causes of artemisinin resistance. Malaria parasites that survive with *Pf*K13 mutations show reduced hemoglobin endocytosis and have lower haem and iron (HI) requirements. We recently showed that malaria parasites depend on HI, and that artemisinin likely kills malaria parasites by disturbing their utilization of HI [19]. The high HI requirement of* Plasmodium* parasites may result in their high sensitivity to artemisinin [19, 20]; thus, there may be a different explanation for artemisinin resistance in *Plasmodium* with *Pf*K13 mutations. However, regardless of which explanation is better supported, it is essential to clarify how *Pf*K13 mutations affect malaria parasites.

## Methods

### In vitro culture of *P. falciparum* parasites and synchronization

*Plasmodium falciparum* 3D7^WT^ and 3D7^C580Y^ were cultured in Roswell Park Memorial Institute (RPMI) 1640 medium with 25 mmol/L HEPES, 0.5% AlbuMAX, 0.2% sodium bicarbonate, 0.2 mmol/L htpoxanthine, and 20 μg/mL gentamicin sulfate at 37 °C in an incubator supplied with 5% CO_2_ and 5% O_2_. Fresh O positive human red blood cells from healthy human donors were used to maintain parasitaemia; the parasites were maintained at 2% hematocrit, based on a previous report [21]. The parasites were synchronized by treatment with 5% sorbitol, incubated at 37 °C for 10 min, washed with RPMI 1640, and then returned to normal culture conditions. Mature schizonts were purified using a 40–70% Percoll gradient, washed with RPMI 1640, and cultured for 3 or 6 h with fresh erythrocytes. The cultures were further treated with 5% sorbitol to obtain 0- to 3 or 0- to 6-h ring stages. Parasitaemia was calculated from Giemsa blood smears observed under a light microscope.

### Sample preparation and RNA isolation

The cultures were tightly synchronized to obtain 0- to 3-h ring stages. Parasites were harvested at 0, 6, 12, 18, 24, 30, 36, and 42 h post-infection (hpi) for transcriptome analysis. Samples at different stages were collected by centrifugation at 1000* g* for 3 min and preserved in TRIzol reagent (Invitrogen, USA). RNA was extracted using phenol and isopropanol precipitation. RNA quantification and quality determination were performed using Nanodrop 2000 (Thermo Fisher Scientific, Waltham, MA).

### Library preparation and RNA sequencing

RNA integrity was assessed using an Agilent 2100 Bioanalyzer (Agilent Technologies, Santa Clara, CA). Libraries were constructed using the TruSeq Stranded mRNA LT Sample Prep Kit (Illumina, San Diego, CA) according to the manufacturer’s instructions. Transcriptome sequencing and analysis were conducted by LC Sciences (Hangzhou, Zhejiang, China). The libraries were sequenced on an Illumina HiSeq X Ten platform, and 150 base pair paired-end reads were generated. About 49.10 million raw reads for each sample were generated. Raw data in FASTQ format were first processed using Trimmomatic, and low-quality reads were removed to obtain the clean reads [22]. About 48.02 million clean reads for each sample were retained for subsequent analyses.

### Differentially expressed gene analysis

Clean reads were mapped to the *P. falciparum* genome by using HISAT2 [23]. The fragments per kilobase of transcript per million mapped reads of each gene was calculated using Cufflinks [24, 25], and the read counts of each gene were obtained using htseq-count [26]. Differential expression analysis was performed using the DESeq (2012) package in R (https://bioconductor.org/packages/release/bioc/html/edgeR.html). The threshold for significant differential expression was set at *P-* < 0.05, fold change > 2, or fold change < 0.5, and the minimum number of reads for at least one sample had to exceed 100. Gene Ontology (GO) enrichment analysis was performed using R or David software (https://david.ncifcrf.gov). Differentially expressed genes were functionally annotated using PlasmoDB and UniProt.

### Sample preparation and mass spectrometry analysis

*Plasmodium falciparum* 3D7^WT^ and *P. falciparum* 3D7^C580Y^ were treated to obtain parasites 0–3 h after invasion, as described above, and samples were harvested at 12, 24, and 30 h. Extraction, purification and mass spectrometry analysis of the protein were performed by LC-Bio Technology (Hangzhou, China) through liquid chromatography–tandem mass spectrometry. During the differentially expressed protein analysis, fold change > 2 or fold change < 0.5 served as the screening criterion. The selected differentially expressed proteins were subjected to GO enrichment analysis.

### Primer design and real-time polymerase chain reaction analysis

Total RNA from *P. falciparum* 3D7^WT^ and *P. falciparum* 3D7^C580Y^ was isolated using TRIzol reagent. Following DNase treatment, total RNA (800 ng) was reverse transcribed into complementary DNA using the ChamQ Universal SYBR qPCR master mix (Vazyme, Nanjing, China) according to the manufacturer’s instructions on a 7500 Real-Time PCR System (Applied Biosystems). We selected genes such as merozoite structure-related genes, merozoite surface protein MSA180 gene, rhoptry protein genes, DNA replication-related genes, DNA replication licensing factor MCM5 gene, and proliferating cell nuclear antigen 1 gene, which showed differential expression in the transcriptomes. Seryl-transfer RNA synthetase served as the internal control, and its expression was measured by detecting the fluorescence from SYBR green dye incorporated into the amplifying target. RNA expression was normalized to the corresponding internal control genes, and relative changes were calculated using the 2^−^$\Delta \Delta$^Ct^ method [27, 28]. The experiment was performed using three independent biological and technical replicates. For primer design, we used Primer BLAST (https://www.ncbi.nlm.nih.gov/tools/primer-blast/). Primers used for quantitative real-time polymerase chain reacion (qPCR) are listed in Additional file 2: Table S1.

### Comparison of growth curves

*Plasmodium falciparum* 3D7^WT^ and *P. falciparum* 3D7^C580Y^ were synchronized several times to obtain 0- to 3-h ring stages. The initial infection rate was maintained at 0.3% with hematocrit at 2%, after which normal culture conditions were used. The parasites were cultured for 144 h, and infected red blood cells were collected every 48 h. These were then incubated with Hoechst 33342 (catalogue no. C1028; Biyuntian) in the dark at 37 °C for 10 min. After two washes with RPMI 1640, positive cells were sorted using the BD FACS AriaII cell sorter based on a 355-nm (Hoechst 33342) fluorescence signal. Uninfected red blood cells were used as controls. All data were processed using FlowJo software (10.8.1).

### Analysis of the number of merozoites

Mature schizonts were synchronized using a 40–70% Percoll gradient and cultured at 37 °C for 3 h with shaking to reduce multiple infections. After synchronization with 5% sorbitol, the parasite was cultured for another 42–45 h. The number of merozoites in the schizonts was counted under light microscopy after Giemsa staining. Data were collected through repeated experiments conducted by three operators.

### Comparison of hemozoin content

*Plasmodium falciparum* 3D7^WT^ and *P. falciparum* 3D7^C580Y^ were purified using a 40–70% Percoll gradient and treated with 5% sorbitol to obtain 6-h ring stages. Then, *P. falciparum* 3D7^WT^ and *P. falciparum* 3D7^C580Y^ were cultured for 36–42 h with parasitaemia adjusted to 0.5–1% by using normal erythrocytes while maintaining 2% hematocrit. Approximately 10 ml of the culture medium was centrifuged at 1000* g* for 3 min, and then treated with 0.15% saponin solution on ice for 10 min to lyse the cells. After centrifugation at 12,000 *g* for 10 min, the supernatant was discarded, and the sediment was washed with 25 mmol/L Tris (pH 7.8) containing 2.5% sodium dodecyl sulphate until the supernatant was clear. The sediment was dissolved in 250 µL of 2.5% sodium dodecyl sulphate buffer and 20 µL of 2.5 mol/L NaOH. The absorbance of hemozoin at 400 nm was measured using a Nanodrop 2000 spectrophotometer to quantify its content [29].

###  In vitro iron sucrose assay

*Plasmodium falciparum* 3D7^WT^ and *P. falciparum* 3D7^C580Y^ were tightly synchronized to obtain the 0- to 3-h ring stages. Synchronized ring-stage parasites were incubated with 50 µmol/L iron sucrose, and the control group was incubated with an equal volume of RPMI 1640 complete medium. After 90–96 h of incubation, the merozoites were counted under a light microscope following Giemsa staining. Data were obtained from experiments repeated at least three times.

### Desferrioxamine treatment

*Plasmodium falciparum* 3D7^WT^ at 1.5–2.5% parasitaemia was treated with desferrioxamine (DFO) (MedChemExpress, HY-B1625) at a final concentration of 500 nmol/L for 24 h. A portion of the mixture was centrifuged at 1000 *g* for 5 min to remove the supernatant. After the addition of cells suspended in PBS and Hoechst 33342 (1:100) (Biyuntian), the mixture was incubated at 37 °C for 10 min under continuous mixing, centrifuged at 1000 *g* for 5 min, and washed three times with phosphate-buffered saline. The positive cell rate was determined using a BD FACS AriaII cell sorter. Infected red blood cells were identified by using 355-nm fluorescence spectroscopy, with uninfected cells serving as controls. All data were processed using FlowJo software (10.8.1).

### Statistical analysis

Graphpad Prism 8.0.2 software was used to perform the statistical analysis. Student's *t*-test (unpaired) was performed to compare the changes in parasitaemia in the groups. *P* < 0.05 was considered to indicate statistical significance.

## Results

### Different effects of *Pf*K13 mutation on the transcriptome and proteome of *P. falciparum* at different stages

**Fig. 1:**
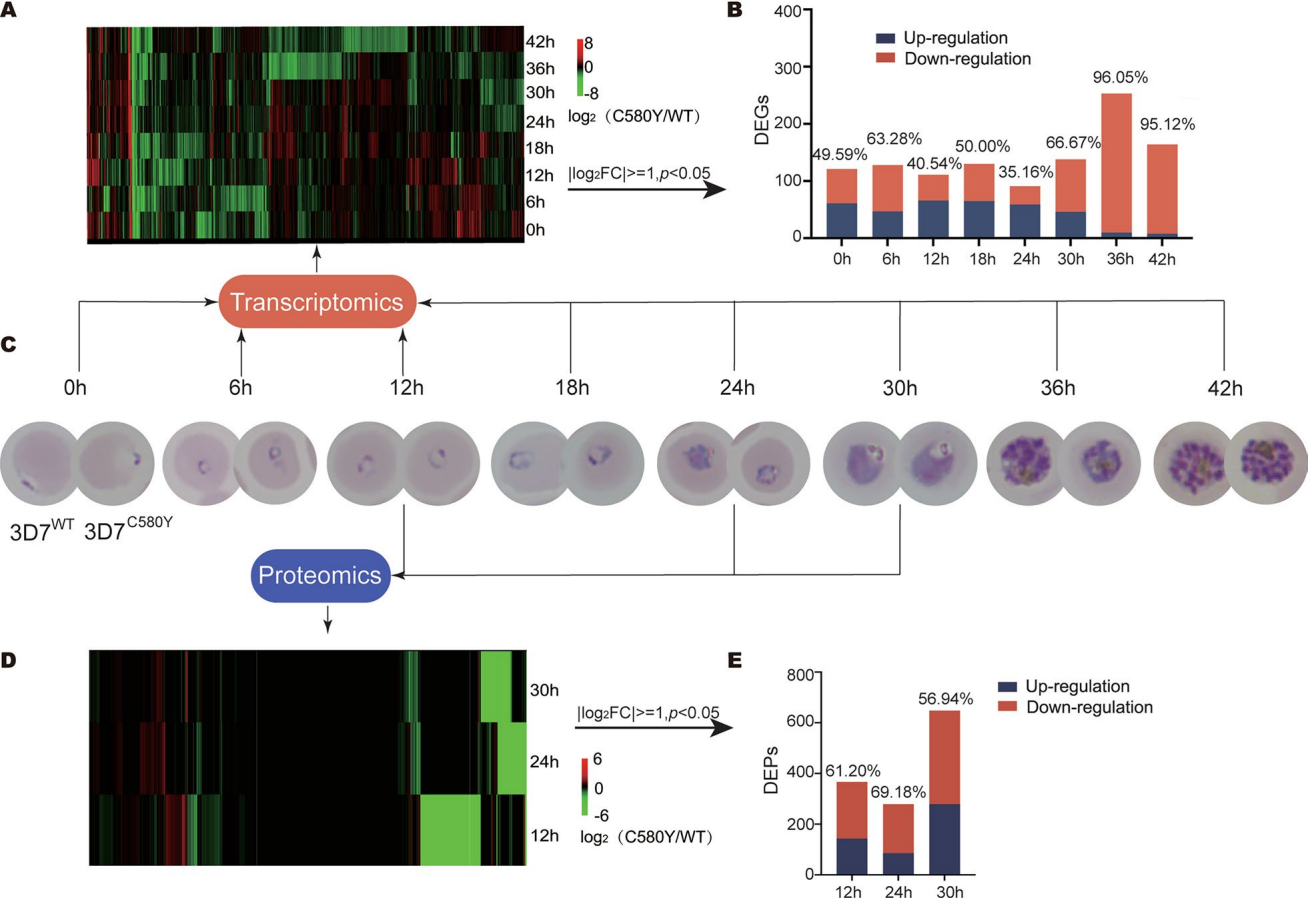
Different effects of *Pf*K13 mutation on the transcriptome and proteome of *Plasmodium falciparum* at different stages. **A** Heat map of all differentially expressed genes (DEGs) (*P. falciparum* 3D7^C580Y^/*P. falciparum* 3D7^WT^). **B** DEGs at different stages (|log_2_ fold change (FC)| ≥ 1, *P* < 0.05; the minimum number of reads for at least one sample had to exceed 100). **C** *Plasmodium falciparum* 3D7^WT^ and *P. falciparum* 3D7^C580Y^ at different stages. **D** Heat map of differentially expressed proteins (DEPs) (*P. falciparum* 3D7^C580Y^/*P. falciparum* 3D7^WT^). **E** DEPs at 12, 24, and 30 h post-infection (hpi) (|log_2_FC| ≥ 1, *P* < 0.05)

The transcriptomes of *P. falciparum* 3D7^WT^ and *P. falciparum* 3D7^C580Y^ were analyzed at 0, 6, 12, 18, 24, 30, 36, and 42 hpi (Fig. 1A–C; Additional file 3: Table S2). Among the 5282 identified genes, 123, 128, 111, 130, 91, 138, 253, and 164 were differentially expressed at various time points post-infection (Additional file 3: Table S2). At 36 and 42 hpi, the downregulated genes (*P. falciparum* 3D7^C580Y^ vs. *P. falciparum* 3D7^WT^) accounted for 96.05% and 95.12% of the differentially expressed genes, respectively (Fig. 1B). Additionally, proteomes of the samples at 12, 24, and 30 hpi were also analyzed. Among all the identified 2558 proteins, 366, 279, and 648 differentially expressed proteins were detected (Additional file 4: Table S3). The downregulated proteins (*P. falciparum* 3D7^C580Y^ vs. *P. falciparum* 3D7^WT^) accounted for 61.20%, 69.18%, and 56.94%, respectively (Figs. 1D, E; Additional file 4: Table S3).

### *Pf*K13 mutation especially decreased the expression of DNA synthesis- and merozoite structure-related genes

**Fig. 2:**
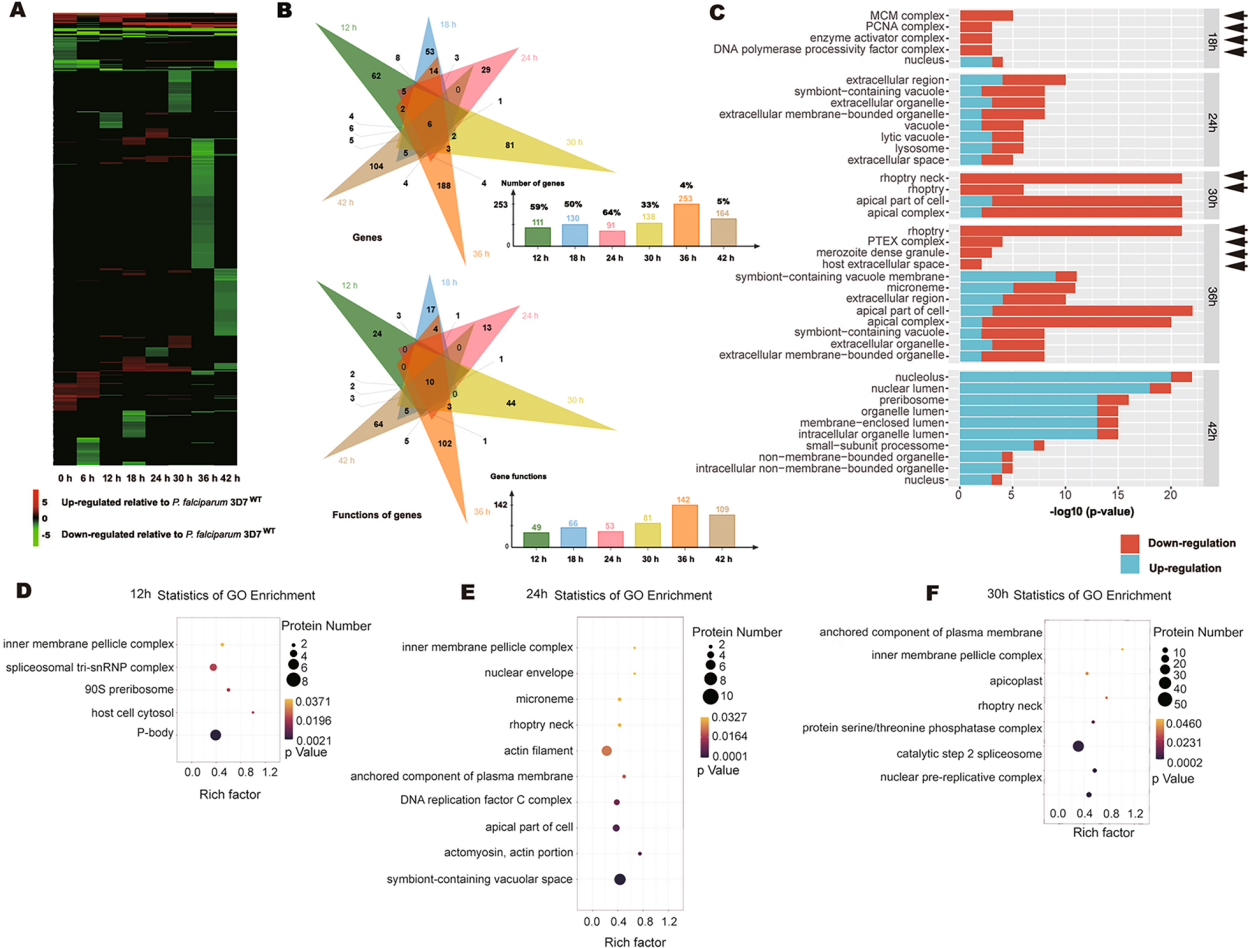
Analysis of DEGs and DEPs and their functions between *Plasmodium falciparum* 3D7^WT^ and *P. falciparum* 3D7^C580Y^. **A** Heat map of DEGs (*P. falciparum* 3D7^C580Y^/*P. falciparum* 3D7^WT^) at different parasite stages. **B** Venn plots of DEGs at 12, 18, 24, 30, 36, and 42 hpi and their functions. The percentages represent the proportion of upregulated DEGs (*P. falciparum* 3D7^C580Y^/*P. falciparum* 3D7^WT^). **C** Gene Ontology (GO) enrichment analysis of DEGs at 18, 24, 30, 36, and 42 hpi. **D**–**F** GO enrichment analysis of DEPs (*P. falciparum* 3D7^C580Y^/*P. falciparum* 3D7^WT^) at 12, 24, and 30 hpi. For other abbreviations, see Fig. 1

The characteristic differentially expressed genes between *P. falciparum* 3D7^C580Y^ and *P. falciparum* 3D7^WT^ at various time points post-infection indicated differences or changes in main functions at these various time points. Especially at 18 hpi, DNA synthesis-related genes and proteins, such as the minichromosome maintenance protein complex, proliferating cell nuclear antigen complex, and DNA polymerase processivity factor complex were downregulated. At 30 h and 36 hpi, all or most merozoite structure-related genes, such as rhoptry, merozoite dense granules, microneme, and apical complex genes were downregulated (Fig. 2A–C). Proteome analysis also showed that at 24 and 30 hpi, the expression of merozoite structure-related proteins was downregulated (Fig. 2D–F), which supported the results of the transcriptome analysis.

### *Pf*K13 mutation did not significantly affect metabolism but did weaken reproduction

**Fig. 3A–H:**
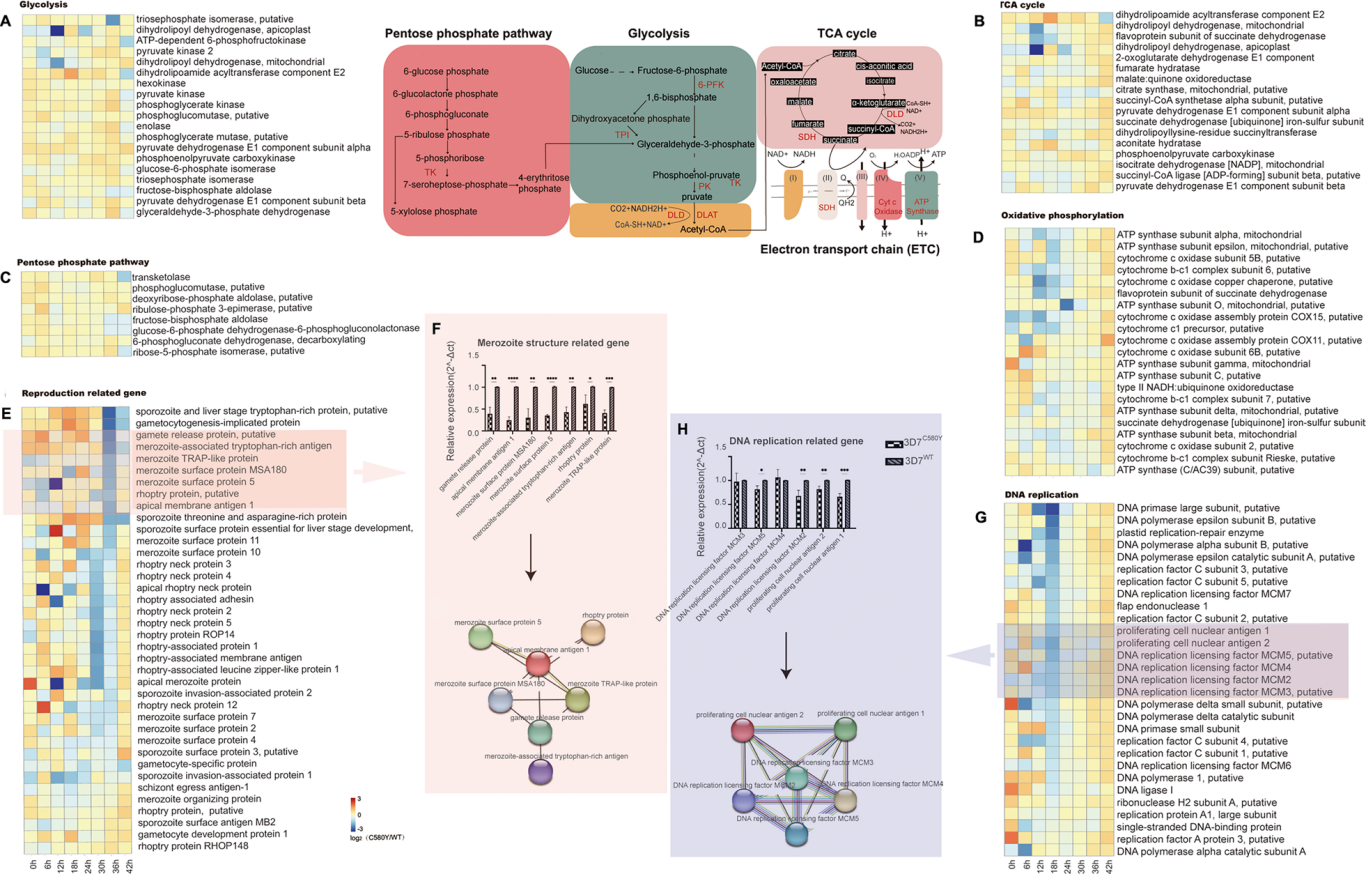
Differential expression of genes associated with glycolysis, the tricarboxylic acid cycle (TCA) cycle, pentose phosphate pathway (PPP), oxidative phosphorylation, reproduction, and DNA replication between *Plasmodium falciparum* 3D7^C580Y^ and *P. falciparum* 3D7^WT^. **A** Heat map of DEGs associated with glycolysis (*P. falciparum* 3D7^C580Y^/*P. falciparum* 3D7^WT^) at different stages. **B** TCA cycle, **C** PPP, **D** oxidative phosphorylation, **E** reproduction-related genes, **F** and validation of reproduction-related genes through real-time polymerase chain reacion (PCR) and their interaction network. **G** Heat map of DEGs associated with DNA replication (*P. falciparum* 3D7^C580Y^/*P. falciparum* 3D7^WT^) at different stages. **H** Validation of DNA replication-related genes through real-time PCR and their interaction network. *P < 0.05, **P < 0.01, ***P < 0.001, ****P < 0.0001

To investigate the effect of *Pf*K13 protein mutation on various metabolic processes in *Plasmodium*, we analyzed the expression of genes associated with glycolysis, the tricarboxylic acid cycle, the pentose phosphate pathway (PPP), oxidative phosphorylation, reproduction, DNA replication, pyrimidine metabolism, fatty acid metabolism, amino acid metabolism, purine metabolism, and redox pathways, including the glutathione and thioredoxin systems (Fig. 3; Additional file 1: Fig. S1), which have often been associated with drug resistance in malaria parasites [30]. The expression of most genes associated with glycolysis, the tricarboxylic acid cycle, the PPP, oxidative phosphorylation, fatty acid metabolism, amino acid metabolism, purine and pyrimidine metabolism, and redox processes was unaffected, that is, most genes were not differentially expressed between *P. falciparum* 3D7^C580Y^ and *P. falciparum* 3D7^WT^. Although a few genes were differentially expressed, some of them were upregulated or downregulated for *P. falciparum* 3D7^C580Y^ and *P. falciparum* 3D7^WT^, and it was difficult to determine if the overall process was being facilitated or inhibited (Fig. 3; Additional file 1: Fig. S1). However, most of the genes associated with merozoites, sporozoites, and gametocytes were downregulated at 30 and 36 hpi (Fig. 3E), and most of the genes associated with DNA replication were downregulated at 18 hpi (Fig. 3G), which is consistent with the findings of a previous study [31]. Some of these results were further validated by real-time PCR, which revealed the close functional relationships of the genes (Fig. 3F, H).

### *P. falciparum* 3D7^C580Y^ had fewer merozoites than *P. falciparum* 3D7^WT^

**Fig. 4:**
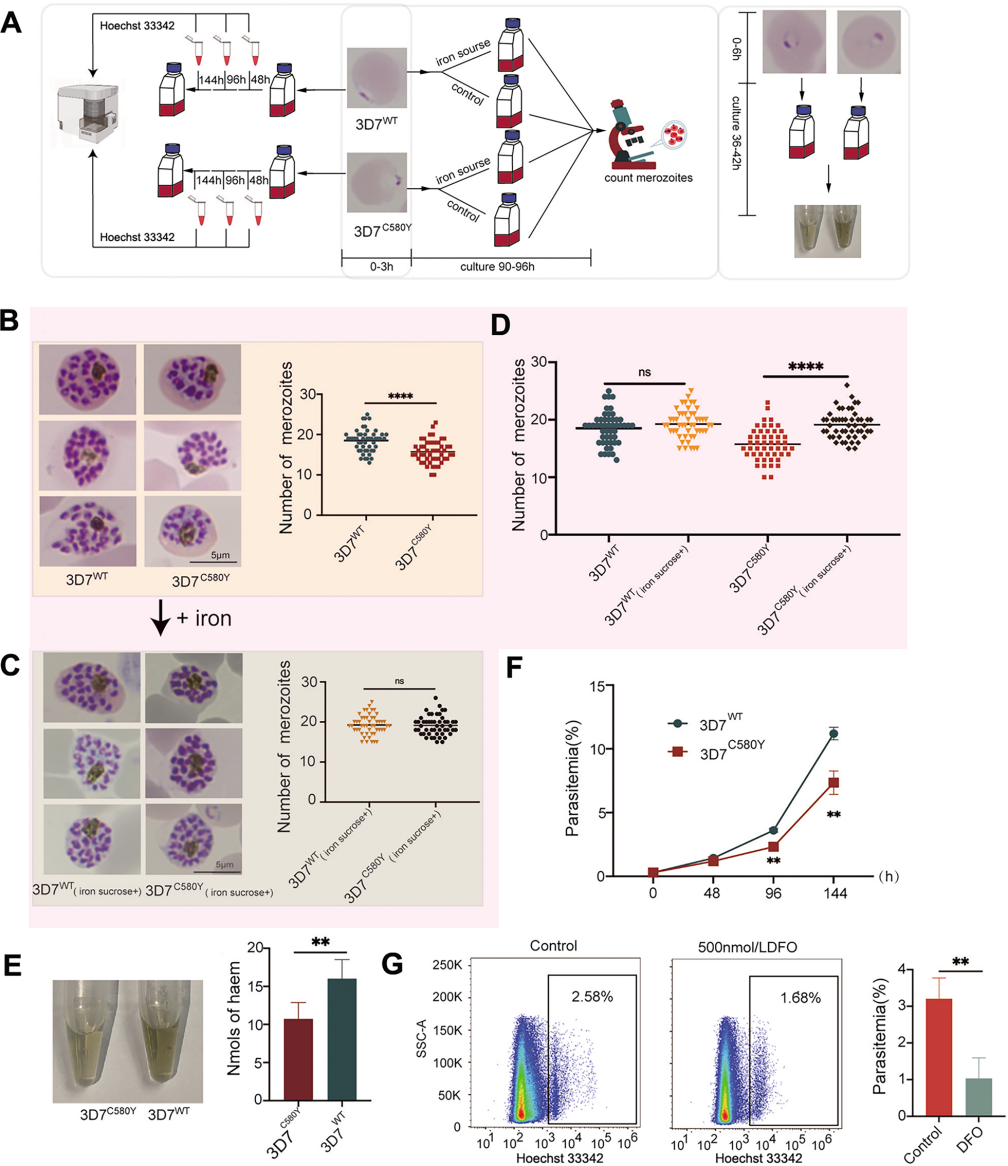
Effect of *Pf*K13 mutation or iron supplementation on the reproduction of *Plasmodium falciparum* 3D7. **A** The workflow involved monitoring growth curves, conducting in vitro iron sucrose assays, and comparing hemozoin content. **B** Comparison of merozoites and their number between *P. falciparum* 3D7^WT^ and *P. falciparum* 3D7^C580Y^. **C** Impact of iron supplementation on merozoites and their number in *P. falciparum* 3D7^C580Y^. **D** Effect of iron supplementation on the number of merozoites of *P. falciparum* 3D7^WT^ and *P. falciparum* 3D7^C580Y^. **E** Comparison of hemozoin content between *P. falciparum* 3D7^WT^ and *P. falciparum* 3D7^C580Y^. **F** Comparison of growth curves between *P. falciparum* 3D7^WT^ and *P. falciparum* 3D7^C580Y^. **G** Flow-cytometry analysis showing the impact of desferrioxamine (DFO) on the growth and development of *P. falciparum* 3D7.* ns* No significant difference (*P* > 0.05), **P < 0.01, ****P < 0.0001 (t-test)

An inhibitory effect of *Pf*K13 mutation on reproduction was revealed. To further confirm this finding, we compared the number of merozoites between *P. falciparum* 3D7^C580Y^ and *P. falciparum* 3D7^WT^. We quantified the number of merozoites in 50 mature schizonts and observed that *P. falciparum* 3D7^C580Y^ had an average of 16 ± 3 merozoites, whereas *P. falciparum* 3D7^WT^ had an average of 19 ± 3 (*P* < 0.0001) (Fig. 4A, B).

### Hemozoin content of *P. falciparum* 3D7^C580Y^ was lower than that of *P. falciparum* 3D7^WT^

Hemozoin, which is considered a vector of HI in hemozoin-forming organisms, functions in reproduction [29, 32]. Hemozoin content was found to correlate with the utilization of HI and artemisinin sensitivity [20]. To investigate the effect of the *Pf*K13 mutation on HI utilization, we compared the hemozoin content of an equivalent number of *P. falciparum* 3D7^C580Y^ and *P. falciparum* 3D7^WT^ at 36 hpi. The hemozoin content of *P. falciparum* 3D7^WT^ was 16.02 ± 2.44 nmol and that of *P. falciparum* 3D7^C580Y^ 10.72 ± 2.13 nmol. The hemozoin content of *P. falciparum* 3D7^C580Y^ was significantly lower than that of *P. falciparum* 3D7^WT^ (*P* < 0.01) (Fig. 4E).

### Growth of *P. falciparum* 3D7^C580Y^ was slower than that of *P. falciparum* 3D7^WT^

After three cycles of culture, the mean parasitaemia of *P. falciparum* 3D7^C580Y^ was 7.35%, which was significantly lower than that observed in *P. falciparum* 3D7^WT^ (11.2%) (*P* < 0.01). This suggested that the growth and reproductive capability of *P. falciparum* 3D7^C580Y^ was lower than that of *P. falciparum* 3D7^WT^ (Fig. 4F).

### Addition of iron increased the number of *P. falciparum* 3D7^C580Y^ merozoites

The previous findings indicate that *P. falciparum* 3D7^C580Y^ had a reduced reproductive capacity and hemozoin content compared with *P. falciparum* 3D7^WT^. To investigate the effect of iron on the reproduction of *P. falciparum* 3D7^C580Y^, we supplemented the culture system with sucrose iron and quantified the number of merozoites after 90–96 h of cultivation. The number of merozoites of *P. falciparum* 3D7^C580Y^ increased significantly from 16 ± 3 to 19 ± 3 (*P* < 0.0001) (Fig. 4C, D), which was similar to the increase seen in *P. falciparum* 3D7^WT^ (Fig. 4D).

### Effect of DFO on *P. falciparum*

Iron supplementation promoted the reproduction of *P. falciparum* 3D7^C580Y^. To further elucidate the role of iron in *Plasmodium* proliferation and development, we treated *P. falciparum* 3D7 with DFO for 24 h and observed its effect on the parasites. The parasitaemia of *P. falciparum* 3D7 decreased from 3.21% ± 0.55% to 1.03% ± 0.56% after treatment with DFO (*P* < 0.01) (Fig. 4G).

## Discussion

**Fig. 5:**
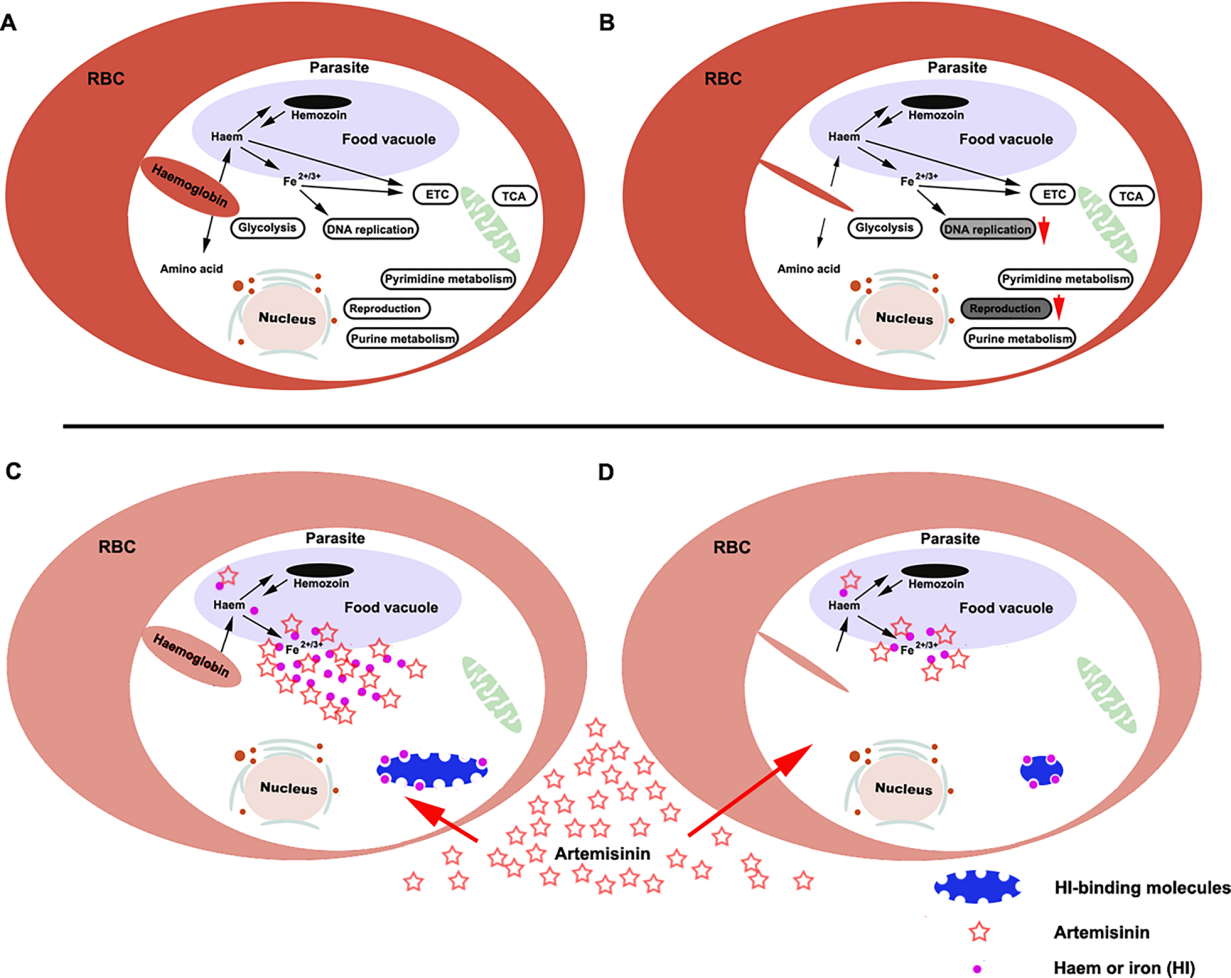
Effect of *Pf*K13 mutation on malaria parasites. **A** The schematic illustrates the primary metabolic pathways following hemoglobin endocytosis in *Plasmodium falciparum* 3D7^WT^. **B** *Pf*K13 mutation attenuated hemoglobin endocytosis without significantly altering the associated primary metabolic pathways while decreasing the expression of genes related to reproduction and DNA synthesis. **C**–**E** The schematic illustrates the two potential mechanisms of artemisinin resistance resulting from *Pf*K13 mutations. The release of haem and iron (HI) after the endocytosis and digestion of hemoglobin has been well established. Upon entering a parasite, artemisinin is activated by HI and simultaneously sequesters HI, resulting in two detrimental effects in the parasites: generation of damage-inflicting free radicals, and disruption of the function of HI-related molecules due to HI deficiency (**C**). On the one hand, *Pf*K13 mutations reduced hemoglobin endocytosis and impaired HI-mediated artemisinin activation, which led to resistance against artemisinin (**D**). On the other hand, the reduced requirement for HI was easily overcome by the presence of a small amount of iron in the parasite, resulting in the resistance of the parasites to the HI deficiency caused by the action of artemisinin (**D**)

The World Health Organization recommends that the presence of persistent parasitaemia on day 3 after treatment with artemisinin should be considered confirmatory for the resistance of* P. falciparum* to this drug. The phenotypic manifestation of artemisinin-resistant *P. falciparum* infections is slowed parasite clearance after treatment with artesunate in vivo. This slow-clearance phenotype, defined by a parasite clearance half-life of > 5 h, is attributed to the loss of sensitivity of *P. falciparum* to artemisinin during the early stage of the intraerythrocytic developmental cycle, the ring stage [33, 34]. Resistance (or tolerance) in cultured parasites is defined as > 1% survival in the ring-stage survival assay, in which parasites at the young ring stage (0–3 hpi) are exposed to 700 nM DHA for 4–6 h, and their survival rate is measured 3 days later [34]. This level of survival is considered to indicate partial resistance to artemisinin or its derivatives. Artemisinin resistance is considered to be associated with the genetic and transcriptomic background or initial transcriptional responses of susceptible parasites to artemisinin [13, 35]. The mechanisms by which *Pf*K13 mutations may lead to resistance are both controversial and interesting. Previous studies have proposed that *Pf*K13 mutations could change various aspects of the parasite’s intraerythrocytic developmental program, by influencing cell-cycle periodicity, unfolded-protein response, protein degradation, vesicular trafficking, DNA replication and repair [8, 30, 31]. Moreover, mitochondrial metabolism or processes including damage sensing and antioxidant properties likely augment the protection of *P. falciparum* with a *Pf*K13 mutation from artemisinin by helping these parasites to undergo temporary quiescence and then recovery through accelerated growth after drug elimination [30]. *Pf*K13 mutation did not significantly influence the main metabolic processes of glycolysis, the TCA cycle, PPP, fatty acid metabolism, pyrimidine metabolism, purine metabolism, antioxidation, or the electron transport chain in the present study; however, DNA replication decreased at 18 hpi. *Pf*K13 mutation also inhibited the expression of merozoite-related genes, which was accompanied by a reduced number of merozoites, slow growth, and decreased hemozoin content in *P. falciparum* 3D7^C580Y^ (Fig. 5A, B). Why the *Pf*K13 mutation affected mostly the expression of DNA replication- and merozoite-related genes is of interest.

Superficially, the *Pf*K13 mutation reduces hemoglobin endocytosis, resulting in decreased amino acid supply and nutrient deficiency, ultimately leading to reduced reproductive ability. Simultaneously, reduced HI release diminishes artemisinin activation, thereby contributing to the development of artemisinin resistance [8]. However, it is worth noting that the *Pf*K13 mutation, which resulted in a reduction of HI release, not only diminished the activation of artemisinin but also decreased the requirement for and utilization of HI (Fig. 5C, D). When insufficient HI are provided to parasites, their functions associated with HI utilization are impaired. When the parasites do not die, their related functions and metabolism will change in accordance with the low level of HI utilized. As the level of utilized HI is low, efficient activation of the pentose phosphate pathway (PPP), which synthesizes pentose phosphate and NADPH and can thus promote DNA synthesis and GSH cycling, is hindered. Consequently, DNA synthesis declines and it becomes difficult to maintain the GSH cycle, which ultimately leads to decreased fertility [19]. Notably, parasites that utilize low levels of HI can achieve greater resistance to HI depletion. A potential mechanism to explain this, that artemisinin kills malaria parasites by disrupting their utilization of HI, is another likely reason why the parasites develop resistance to artemisinin [19]. This theory suggests that HI use decreases with a decreased HI requirement, leading to artemisinin-induced parasite resistance to the exhaustion of HI.

HI are crucial for the reproduction and development of malaria parasites [20, 36–39]. DNA synthesis-related enzymes in particular require an iron–sulfur cluster to form active complexes [40–45]. In our experiment, iron supplementation alone restored the number of merozoites of *P. falciparum* 3D7C^580Y^ to the normal level, which indicated that the *Pf*K13 mutation likely weakens reproductive ability by reducing hemoglobin endocytosis to decrease the HI supply. In fact, *Pf*K13 mutation, on the one hand, can lead to a reduction in the supply of amino acids and HI, and thus delay development and reduce the activation of artemisinin. On the other hand, the parasites can become resistant to artemisinin as a consequence of the *Pf*K13 mutation leading to a reduction in their requirement for and dependence on HI [19]. Determining which is the dominant mechanism, or whether both mechanisms operate concurrently, is of interest.

Given that the parasites were alive after the *Pf*K13 mutation arose, the HI requirement of *P. falciparum* 3D7^C580Y^ was reduced compared with that of *P. falciparum* 3D7^WT^. Our findings revealed the differential expression of some genes associated with glycolysis, the TCA cycle, PPP, fatty acid metabolism, antioxidation, and the electron transport chain between *P. falciparum* 3D7^C580Y^ and *P. falciparum* 3D7^WT^. However, regular expression patterns were not observed, unlike in DNA synthesis-related or reproduction-related genes that were downregulated in *P. falciparum* 3D7^C580Y^, which clearly indicated that *Pf*K13 mutation primarily affected HI supply. Krugliak et al. [46] proposed that the parasite digests up to 65% of the host cell's hemoglobin but utilizes only up to approximately 16% of the amino acids derived from it. This large discrepancy indicates that the parasites prefer HI to amino acids present in the host hemoglobin. The *Pf*K13 mutation may primarily lead to a decrease in the uptake of iron rather than that of amino acids in the parasites.

In the present study, the number of merozoites of *P. falciparum* 3D7^C580Y^ was restored to a level similar to that of *P. falciparum* 3D7^WT^ simply by iron supplementation. Furthermore, when iron was removed by using DFO, *P. falciparum* 3D7^WT^ was inhibited or killed. These results confirm that iron is crucial to the parasite. Therefore, the question arises as to whether *Pf*K13 mutation confers resistance by reducing the activation of artemisinin or by decreasing HI requirements.

In brief, although reduced activation of artemisinin due to a reduction in hemoglobin digestion and a reduced level of HI can logically explain resistance to artemisinin, it is a personalized mechanism that arises as a consequence of *Pf*K13 mutation. It is difficult to base an explanation for artemisinin resistance on other mutations. However, a different mechanism through which the HI requirement of *P. falciparum* 3D7^C580Y^ is decreased, which results in artemisinin resistance, is useful for elucidating other mutations that are responsible for resistance to artemisinin or identifying organisms that are insensitive to this drug.

### Supplementary Information

**Additional file 1: Figure S1**. Differential expression of genes associated with pyrimidine metabolism, fatty acid metabolism, amino acid metabolism, purine metabolism, and redox processes between *Plasmodium falciparum* 3D7C580Y and *P. falciparum* 3D7WT. **A** Heat map of differentially expressed genes associated with pyrimidine metabolism (*P. falciparum* 3D7C580Y/*P. falciparum* 3D7WT) at different stages, **B** fatty acid metabolism, **C** amino acid metabolism, **D** purine metabolism, and **E** redox processes.

**Additional file 2: Table S1**. Primer sequences of key target genes.

**Additional file 3: Table S2**. Analysis of differentially expressed genes at different stages of the parasite.

**Additional file 4: Table S3**. Analysis of differentially expressed proteins at 12, 24, and 30 h post-infection.

## Data Availability

All data generated or analyzed during this study are included in the article.

## Abbreviations

*Pf*K13 mutation: Plasmodium falciparum Kelch 13 protein mutation
TCA cycle: Tricarboxylic acid cycle
PPP: Pentose phosphate pathway
HI: Haem and iron
hpi: Hour post-infection
MS: Mass spectrometry
DFO: Desferrioxamine
MCM proteins: Mini-chromosome maintenance proteins
PCNA: Proliferating cell nuclear antigen
iRBCs: Infected red blood cells
WHO: World health organization
ART: Artemisinin
IDC: Intraerythrocytic developmental cycle
DHA: Dihydroartemisinin
NADPH: Nicotinamide adenine dinucleotide phosphate
GSH: Glutathione
