# Supplementary figures and images for: What exactly does the PfK13 C580Y mutation in Plasmodium falciparum influence?
Source: Parasit Vectors. 2023 Nov 16;16:421. doi: 10.1186/s13071-023-06024-4 (PMC10652512; doi:10.1186/s13071-023-06024-4)

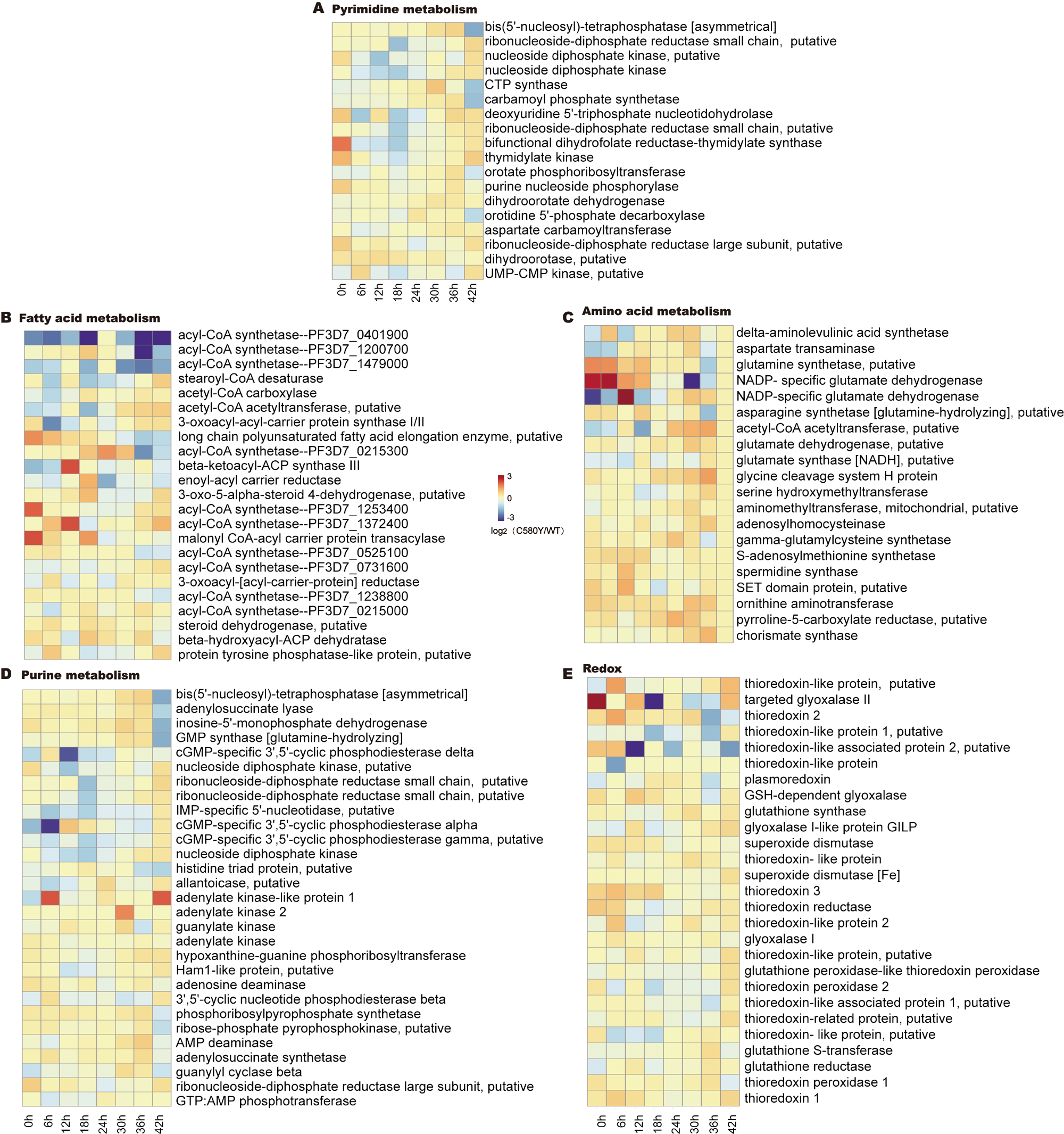

Supplement: Supplementary file 1 — Additional file 1: Figure S1. Differential expression of genes associated with pyrimidine metabolism, fatty acid metabolism, amino acid metabolism, purine metabolism, and redox processes between Plasmodium falciparum 3D7C580Y and P. falciparum 3D7WT. A Heat map of differentially expressed genes associated with pyrimidine metabolism (P. falciparum 3D7C580Y/P. falciparum 3D7WT) at different stages, B fatty acid metabolism, C amino acid metabolism, D purine metabolism, and E redox processes. [file 13071_2023_6024_MOESM1_ESM.tif]
